# Response Surface Methodology–Based Optimization of Localized Heat Treatment for Managing *Cimex lectularius* (Hemiptera: Cimicidae) Infestations

**DOI:** 10.1155/vmi/5596375

**Published:** 2026-05-25

**Authors:** Fitsum Dejene, Solomon Abera, P. R. Jeyaramraja, Yonas Syraji

**Affiliations:** ^1^ Department of Biology, College of Natural Sciences, Arba Minch University, Arba Minch, Ethiopia, amu.edu.et; ^2^ PG and Research Department of Botany, PSG College of Arts & Science, Civil Aerodrome Post, Coimbatore, 641014, India

**Keywords:** bed bug management, *Cimex lectularius*, infestation control, lethal temperature, lethal time

## Abstract

This study explores the efficacy of localized heat treatment as a method for controlling bed bug (*Cimex lectularius* L.) infestations. Experiments were carried out to determine the lethal temperature and time necessary to achieve mortality in bed bugs and their eggs. Various combinations of lethal temperatures and exposure times were applied. A temperature of 45°C resulted in the highest mortality rates, establishing it as a promising pest control approach. The lethal temperatures (36°C–49°C) and times (20–100 min) were optimized using response surface methodology with a central composite design. The study showed that maximum mortality of bed bugs and their eggs was achieved at 45°C with a 60‐min exposure time (100 ± 0%). Additionally, lethal temperature and time were evaluated for infested cracked wood and mattress surfaces. For these surfaces, a lethal temperature of 75°C and an exposure time of 30 min were identified. These findings highlight the potential of heat treatment as an effective pest management strategy, accelerating the elimination of this persistent pest from infested homes.

## 1. Introduction

Bed bug infestations have been increasing worldwide over the past few decades, affecting homes, apartments, hotels, and even public transport [1]. The resurgence of bed bugs is attributed to factors such as insecticide resistance, global travel, and inadequate pest management practices. As noted by Wang and his colleagues, bed bugs (*Cimex lectularius* L.) have become a significant problem in the United States [2]. Several researchers have reported that the distribution and intensity of bed bug infestations have reemerged in Southeast Asia, Japan, and mainland Europe [3–5].

Insecticides are frequently suggested as a primary method for fully eliminating bed bugs. It has become evident in the United States that bed bugs are becoming resistant to commonly used insecticides (pyrethroids) [6, 7]. Direct application of insecticides to mattresses or upholstered furniture poses significant risks of exposing humans to pesticides. As a result, nonchemical approaches and integrated pest management (IPM) strategies have gained popularity for eradicating bed bugs. Bed bugs can be controlled using various nonchemical tools and techniques. Nonchemical methods, such as mattress encasements, hot steam, containerized heat treatments, whole‐house heat treatments, laundering, and freezing, have gained increased recognition [8, 9].

Heat treatment is a nonchemical control method that has gained popularity for managing various pests, including those that infest stored food products and wood structures [10–12]. Given bed bugs’ sensitivity to high temperatures, heat treatment has become a practical and effective method for controlling infestations [13]. According to a study by Pereira and colleagues, mortality of adult bed bugs occurred within a temperature range of 41°C–49°C, with exposure times ranging from 2 to 7 h. Higher temperatures resulted in shorter exposure times required to cause mortality [14]. Kells and Goblirsch also reported that the lethal temperature for adult bed bugs is 48.3°C, while the lethal temperature for bed bug eggs is 54.8°C. They also reported that adult bed bugs exposed to 45°C had a lethal time of 94.8 min [15]. The professional pest management industry typically uses heat treatment temperatures between 45°C and 52°C to effectively kill bed bugs [9, 15].

Although using high temperatures to control bed bug infestations is not a new concept, recent advancements in commercial equipment have made this method more accessible and practical for widespread use [9]. Heat treatments can be carried out in controlled environments such as insulated boxes and heated shipping trailers, or by applying heat directly to rooms and their contents within a building. This approach is referred to as whole‐room heat treatments. Regardless of the method employed, heat treatments provide advantages when incorporated into an IPM plan. The benefits of heat treatments include reduced reliance on insecticides in living spaces, quicker results, the capability to treat a variety of items, and less effort required to handle different belongings such as infested laundry and electronics [15].

Insects can only function and develop within a specific temperature range, and exposure to temperatures outside this range first causes immobilization, followed by a coma, and ultimately death. There are a few species of insects that can tolerate body freezing; however, most cannot [16–18]. Temperature shifts disrupt enzymatic activity and insect metabolism, ultimately leading to mortality [19, 20].

Our study aimed to bridge the knowledge gap in the national context by offering the first comprehensive investigation into the efficacy of heat treatment therapy for managing bed bug infestations. We employed response surface optimization through a central composite design (CCD) to determine the most effective combinations of lethal temperature and exposure time for bed bug (*C. lectularius* L.) control. Additionally, our research considered the implications of different surfaces, such as cracked wood and mattresses, further contributing to the development of tailored, efficient, and environmentally friendly pest management strategies. This innovative approach aims to advance bed bug control practices and address the challenges posed by these persistent pests.

## 2. Materials and Methods

### 2.1. Study Area, Period, and Population

This research was conducted in Arba Minch, the administrative center of the Gamo Zone in Southern Ethiopia. Geographically situated at 6°02′N and 37°33′E, Arba Minch is located approximately 504 km south of Ethiopia’s capital, Addis Ababa. The municipality encompasses an area of approximately 2184 ha. The region is characterized by a semiarid climate with an average annual temperature of 29.7°C and a mean annual rainfall of approximately 900 mm, which influences the local ecological and microbial dynamics [21, 22].

### 2.2. Study Design

**FIGURE 1:**
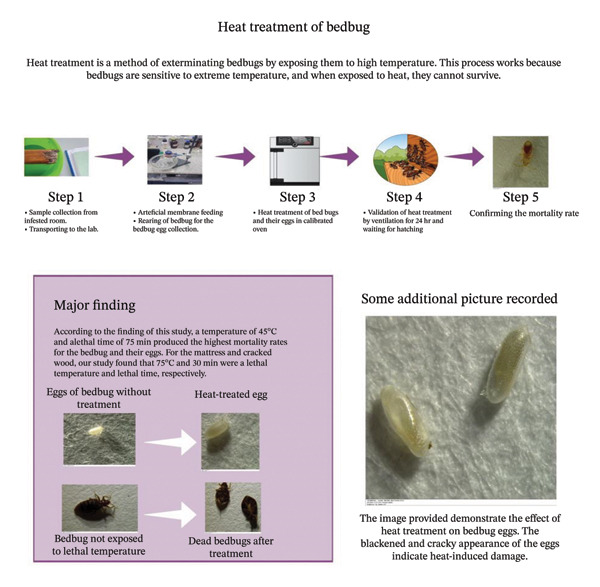
Infographic representation of the study workflow.

Bed bugs were collected using the visual inspection method. The primary eligibility criterion was that the collected samples include various life stages (e.g., adults, nymphs, and eggs) to ensure that the results were representative of the entire population. The second criterion was to exclude bed bugs previously exposed to pesticides, insecticides, or other chemicals that could affect their health or behaviors. Additionally, injured bed bugs or those with visible wounds were excluded to avoid bias from preexisting health conditions. Samples meeting these criteria were collected from Arba Minch town. Forceps or tweezers were used to carefully transfer individual bed bugs into rearing jars or similar containers for transportation and experimentation. An infographic representation of the study workflow is presented in Figure 1.

### 2.3. Selection of Infested Room

According to Singh [23], inspections were performed across the building to identify apartments infested with bed bugs. Climb Up Insect Interceptors were installed under the legs of beds and furniture and inspected after 12–15 days after placement. After inspecting the interceptors, a thorough visual inspection was conducted in rooms confirmed to be infested with bed bugs or suspected of infestation based on history or resident reports. Visual inspections provided accurate estimates of bed bug population levels in units detected by interceptors and identified additional infested units missed by the interceptors.

### 2.4. Sample Collection

In accordance with standard protocol, bed bugs were collected from multiple infested sites, such as apartments, homes, and institutions, in the town of Arba Minch, Gamo Zone. The insects were kept in 240‐mL glass rearing jars, each containing 90‐mm filter paper circles (Whatman No. 1) and fan‐shaped cardboard harborages. The mouth of each rearing jar was sealed with nylon mesh, having 90‐μm openings, to prevent the insects from escaping [14]. An artificial feeding method was used weekly to feed the bed bugs until they were fully engorged [24].

Kells and Goblirsch [15] placed 10 engorged adult bed bugs in 6‐mL plastic vials, which contained several 1 × 3 cm filter paper strips to serve as harborage and oviposition sites. Consequently, the ratio of females to males in the vials was intentionally skewed toward females. The vials were kept under the same conditions as the colony. After 5 days, viable eggs, recognizable by their smooth, white appearance, were collected and used in the experiments without delay [15].

### 2.5. Determination of Lethal Temperature

A hot air oven (MODEL‐CLE‐101 COSLAB, ISO 9001‐2000CO) was used to gradually raise the temperature of a Petri dish containing fed or unfed adult bed bugs or eggs at a steady rate of 0.06°C/min, starting from 20°C. Temperatures of 41, 43, 45, 47, and 49°C were tested using 13 Petri dishes containing adults and 13 petri dishes containing eggs. The experiments were conducted in six trials for adults and three trials for eggs, due to the large number of individuals required.

Control petri dishes containing adults (*n* = 13) or eggs (*n* = 13) were simultaneously placed in a controlled environment at room temperature (23°C). After reaching the target treatment temperature, the designated Petri dish was removed from the oven, along with the corresponding control petri dish from the controlled environment. The Petri dishes were subsequently maintained at 23°C for 24 h, and adult mortality was recorded thereafter.

Adult mortality was determined by the lack of movement when gently touched with forceps. Egg mortality was assessed by the absence of live nymphs hatching, with observations made after 2 weeks.

### 2.6. Determination of Lethal Time at Set Temperatures

Optimal lethal times were determined using response surface methodology (RSM) [25–27]. We employed a CCD, which distributes factor levels across factorial points, center points, and axial (star) points to effectively map the response surface. The Box–Wilson procedure, also referred to as CCD, was used to gather maximum information with the fewest experiments. This study utilized a central composite face‐centered (CCF) experimental design to determine the ideal conditions for lethal time at set temperatures. The multivariate approach allowed for the identification of interactions between variables and a detailed exploration of the experimental domain.

The behavior of the system was represented by the following empirical second‐order polynomial model equation:

(1) Y=β0+∑i=1kβixi+∑i=1kβiixi2+∑i=2k−1∑j=2kβijxixj+ε,

where *Y* represents the predicted response, *x*_*i*_, *x*_*j*_, …, *x*_*k*_ are the input variables affecting *Y*, $x_{i}^{2}$, $x_{j}^{2}$, …, $x_{k}^{2}$ are the squared effects, *x*_*i*_ *x*_*j*_, *x*_*i*_ *x*_*k*_, and *x*_*j*_ *x*_*k*_ are the interaction effects, *β*_0_ is the intercept term, *β*_*i*_ (*i* = 1, 2, …, *k*) represents the linear effect, *β*_*i* *i*_ (*i* = 1, 2, …, *k*) is the squared effect, *β*_*i* *j*_ (*i* = 1, 2, …, *k*; *j* = 1, 2, …, *k*) represents the interaction effect, and *ε* is the statistical error.

### 2.7. Lethal Temperature and Time for Mattress Surface and Wood Crack

According to Wang and his colleagues [8], two wooden boards measuring 8 cm in length, 4 cm in width, and 1 cm in height were constructed. Their design was slightly modified by creating a wedge‐shaped crack in each board to facilitate bed bug infestation. For the treatment, 20 bed bugs were placed on a piece of fabric positioned 5 cm below the upper opening of the wedge‐shaped crack, and 20 eggs were placed on a paper harborage located 6 cm below the opening. Heat treatment was applied immediately after introducing the insects. As a control, additional cracks containing bed bugs and eggs were established but did not receive heat treatment [13].

In addition to testing the effectiveness of heat treatment in wooden cracks, the study evaluated its performance on mattress surfaces. A miniature mattress (measuring 5 cm in length, 5 cm in width, and 3 cm in height) was placed on a plastic tray, and 20 bed bugs were released onto the mattress 1 h before treatment. Furthermore, 20 eggs and a piece of fabric were affixed to the mattress surface just prior to treatment. During the heat treatment, the edge of the treatment chamber was positioned approximately 1 cm from the mattress. A separate mattress containing bed bugs and eggs was prepared as a control, but it was not exposed to heat treatment [13].

Following treatment, the fabric with bed bug eggs was moved to petri dishes and incubated at 26 ± 1°C. Each petri dish lid had a 1.5‐cm‐diameter screened area for ventilation. Egg mortality was assessed after 14 days, with unhatched eggs classified as dead. The bed bugs and eggs from each arena were separately transferred into Petri dishes with a piece of filter paper at the bottom and incubated at 26 ± 1°C. Bed bug mortality was evaluated after 1 day, with those that remained immobile when touched with delicate forceps classified as dead [13].

### 2.8. Statistical Analysis

After each experiment was completed, the data were reviewed, cleaned, and coded for completeness before being entered into Microsoft Excel 2010 and exported to Design‐Expert 11 for formal statistical analysis. Data normality was first assessed using the Shapiro–Wilk test. Because the data were normally distributed, descriptive statistics for bed bug and egg mortality rates are presented as means and standard deviations (SDs). To determine the lethal temperature for bed bugs and eggs, a one‐way analysis of variance (ANOVA) was conducted, followed by Tukey’s post hoc test for multiple pairwise comparisons between temperature groups. For the RSM, multiple regression analysis was performed, and the adequacy of the quadratic models was evaluated using an ANOVA and a lack‐of‐fit test. All hypothesis tests performed were two‐sided, with an a priori level of significance set at *α* = 0.05.

## 3. Results

### 3.1. Determination of Lethal Temperature for Bed Bugs

The lethal effect of temperature on bed bug species was determined by the presence or absence of movement after prodding with forceps. The results shown in Table 1 indicate that temperatures of 41, 43, 45, 47, and 49°C had lethal effects on all stages of the bed bug life cycle. During the experiment, significant knockdown and mortality were observed, with knockdown percentages ranging from 40% to 100% and mortality percentages ranging from 10% to 100%.

**TABLE 1:** Initial knockdown and 24‐h mortality of bed bug adults exposed to five temperatures for varying durations.

Temp (°C)	Time^a^ (min)	% Knockdown^b^ (mean ± SD)	(%) Mortality^c^ (mean ± SD)
41	20	40 ± 10	10 ± 0
	40	60 ± 5.77	30 ± 10
	60	70 ± 5.77	50 ± 5.77
	80	90 ± 10	70 ± 5.77
	100	90 ± 0	80 ± 5.77

43	20	100 ± 0	40 ± 0
	40	100 ± 0	70 ± 0
	60	100 ± 0	80 ± 10
	80	100 ± 0	80 ± 0
	100	100 ± 0	90 ± 5.77

45	20	100 ± 0	60 ± 5.77
	40	100 ± 0	70 ± 0
	60	100 ± 0	100 ± 0
	80	100 ± 0	100 ± 0
	100	100 ± 0	100 ± 0

47	20	100 ± 0	80 ± 5.77
	40	100 ± 0	100 ± 0
	60	100 ± 0	100 ± 0
	80	100 ± 0	100 ± 0
	100	100 ± 0	100 ± 0

49	20	100 ± 0	100 ± 0
	40	100 ± 0	100 ± 0
	60	100 ± 0	100 ± 0
	80	100 ± 0	100 ± 0
	100	100 ± 0	100 ± 0

23	20	0 ± 0	0 ± 0
	40	0 ± 0	0 ± 0
	60	0 ± 0	0 ± 0
	80	0 ± 0	0 ± 0
	100	0 ± 0	0 ± 0

*Note:* The letters a, b, and c are used as footnotes to define the experimental parameters and statistical measures in the table.

**FIGURE 2:**
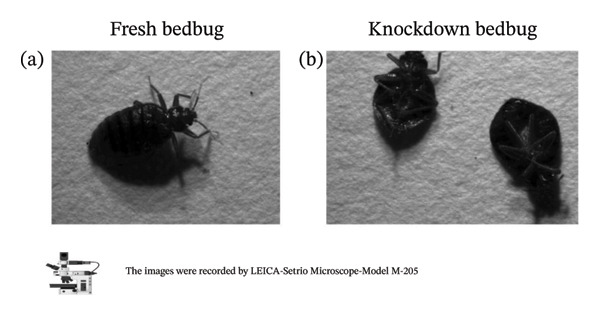
Final knockdown images of bed bugs exposed to lethal temperatures and times. (a) Adult bed bugs; (b) third instars and adult bed bugs. Images were captured using a LEICA M205C stereo microscope.

As shown in Figure 2, all bed bug life stages were exterminated at temperatures in descending order of 49, 47, 45, 43, and 41°C. Among these, 45°C was identified as the optimum temperature, achieving 100% mortality. Bed bugs exposed to heat experienced high levels of knockdown even at lower temperatures. These results indicate that short exposures to temperatures above 41°C can temporarily inactivate bed bugs, even if the temperature does not reach lethal levels. Unfed and fed bed bugs had similar lethal temperatures, so the data were combined.

When mortality across bed bug life stages at different lethal temperatures was compared, statistically significant differences were observed between groups (*F* (5, 24) = 26.058, *p* < 0.001). The Tukey post hoc test revealed no statistically significant differences between the tested temperature groups (*p* > 0.05), except when compared to the control group (*p* < 0.05). Additionally, all temperature groups, except for 43°C, showed statistically significant differences when compared to 41°C (*p* < 0.05).

### 3.2. Determination of Lethal Temperature for Bed Bug Eggs

**FIGURE 3:**
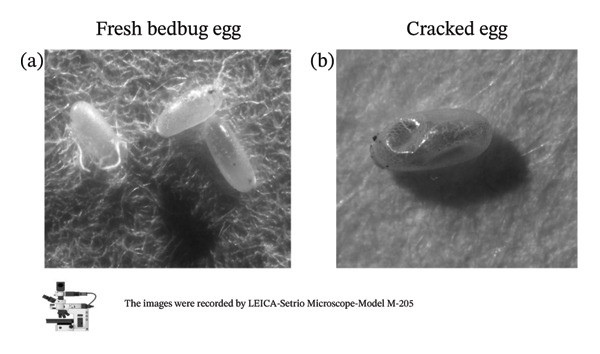
Final cracked images of bed bug eggs exposed to lethal temperatures and times. (a) Untreated image of bed bug eggs; (b) treated images of bed bug eggs. Images were captured using a LEICA M205C stereo microscope.

Table 2 provides a detailed summary of the lethal effects of temperature on bed bug eggs. The selected temperatures were demonstrated to be effective in exterminating the eggs of the common bed bug, *C. lectularius*. Exposure of bed bug eggs to 23°C for up to 100 min caused no mortality. However, survival rates decreased as exposure times to temperatures between 41°C and 49°C increased. For instance, at 41°C, 20 ± 0% mortality was observed after 20 min, and most eggs (80 ± 0%) were killed after 100 min. At 45°C, 60 ± 0% mortality occurred after 20 min, 100 ± 0% after 60 min, and 100 ± 0% after 100 min. Temperatures of 47°C and 49°C caused 100% mortality within just 20 min, as shown in Figure 3.

**TABLE 2:** Mortality of bed bug eggs at different temperatures.

Temp (°C)	Time^a^ (min)	(%) Mortality^c^ (mean ± SD)
41	20	20 ± 0
	40	20 ± 5.77
	60	30 ± 5.77
	80	60 ± 5.77
	100	80 ± 0

43	20	30 ± 0
	40	60 ± 5.77
	60	80 ± 5.77
	80	90 ± 0
	100	90 ± 5.77

45	20	60 ± 0
	40	70 ± 0
	60	100 ± 0
	80	100 ± 0
	100	100 ± 0

47	20	80 ± 5.77
	40	100 ± 0
	60	100 ± 0
	80	100 ± 0
	100	100 ± 0

49	20	100 ± 0
	40	100 ± 0
	60	100 ± 0
	80	100 ± 0
	100	100 ± 0

23	20	0 ± 0
	40	0 ± 0
	60	0 ± 0
	80	3.33 ± 3.33
	100	0 ± 0

*Note:* The letters a, b, and c are used as footnotes to define the experimental parameters and statistical measures in the table.

When mortality rates of bed bug eggs at different lethal temperatures were compared, statistically significant differences were observed between groups (*F* (5, 24) = 23.595, *p* < 0.001). The Tukey post hoc test revealed no statistically significant differences between temperature groups (*p* > 0.05), except when compared to the control group (*p* < 0.05). Additionally, all temperature groups, except for 43°C, showed statistically significant differences when compared to 41°C (*p* < 0.05).

### 3.3. RSM Approach for Bed Bug and Bed Bug Egg Lethal Time Estimation

Table 3 presents the results of experiments using a CCD, which examined the effects of two independent variables on bed bug and egg mortality. Both the expected and actual responses are included. As shown in Table 3, Runs 3, 5, 7, 8, 10, and 11 achieved the maximum percentage mortality, with 100% mortality for both bed bugs and their eggs.

**TABLE 3:** Experimental design and results of the central composite design.

Std	$L_{\text{temp}}^{*}$ (°C)	$L_{\text{Time}}^{*}$ (min)	*L*_temp_ (°C)	*L*_time_ (min)	Response activity (%)			
					Predicted^∗^	Actual^∗^	Predicted	Actual
1	40	20	42	20	59.70	60	61.01	60
2	45	20	48.5	20	81.44	80	87.99	90
3	50	60	55	60	100	100	99.66	100
4	40	100	42	100	85.53	85	79.34	80
5	45	60	48.5	60	97.76	100	99.14	100
6	45	60	48.5	60	97.76	95	99.14	100
7	50	100	55	100	99.70	100	99.34	100
8	45	60	48.5	60	97.76	100	99.14	100
9	45	60	48.5	60	97.76	95	99.14	95
10	45	100	48.5	100	99.77	100	100	100
11	45	60	48.5	60	97.76	100	99.14	100
12	50	20	55	20	88.86	90	91.01	90
13	40	60	42	60	79.77	80	74.66	75

*Note:* $L_{\text{temp}}^{*}$, lethal temperature for the mortality of bed bugs; $L_{\text{Time}}^{*}$, lethal time for the mortality of bed bugs; *L*_temp_, lethal temperature for the mortality of bed bug eggs; *L*_Time_, lethal time for the mortality of bed bug eggs. The asterisk (∗) is used to differentiate between two experimental sets. Columns marked with an asterisk represent the initial predicted and actual values, while unmarked columns represent the values from the second/optimized experimental set.

### 3.4. Model Developed for Estimating the Percentage Mortality

The actual and predicted percentage mortality responses were analyzed using two independent variables. Based on multiple regression analysis of the observed responses, the following quadratic equations were developed:

(2) Y1=+97.7610.839.173.757.167.16+A+B−AB−A2−B2,

(3) Y2=+99.1412.506.672.5011.984.48+A+B−AB−A2−B2,

where *Y*_1_ represents bed bug percentage mortality and *Y*_2_ represents bed bug egg percentage mortality.

The variables *A* and *B* denote the coded values of lethal temperature and lethal time, respectively. The coded equation facilitates response predictions for given factor levels and allows for comparing factor coefficients to assess their relative impacts.

ANOVA results, summarized in Table 4, indicate that bed bug and bed bug egg percentage mortality are influenced by these two parameters. The *R*‐squared values for bed bug and bed bug egg mortality were 0.9794 and 0.9849, respectively, with adjusted *R*‐squared values of 0.9646 and 0.9741, indicating a strong correlation between observed and predicted values and demonstrating a remarkably large effect size, as the treatments account for nearly all the variance in mortality. The predicted *R*‐squared values (0.9428 for bed bugs and 0.9381 for eggs) were consistent with these metrics, confirming the model’s reliability.

**TABLE 4:** Fit statistics for response parameters.

Model developed for design space	*R* ^2^	(Adj. *R* ^2^)^a^	(Pred. *R* ^2^)^b^	Adequate precision	SD^c^	CV^d^
Bed bug	0.9794	0.9646	0.9428	26.9871	2.28	2.50
Bed bug egg	0.9849	0.9741	0.9381	29.1442	2.04	2.22

^a^Adjusted *R*‐squared.

^b^Predicted *R*‐squared.

^c^SD, standard deviation.

^d^CV, coefficient of variation.

A signal‐to‐noise ratio greater than four indicates adequate precision. Here, signal‐to‐noise ratios of 26.987 for bed bug mortality and 29.144 for egg mortality further affirm the experiments’ precision. Table 5 highlights the statistical significance of the quadratic model, with *F*‐values of 66.43 for bed bugs and 91.18 for eggs. All main effects (*A*, *B*) and quadratic effects (*A* ^2^, *B* ^2^) were significant, alongside the interaction term (*AB*). Additionally, the lack‐of‐fit test was not significant (*p* > 0.05), validating the quadratic model for estimating percentage mortality.

**TABLE 5:** ANOVA results for response variables.

Model developed for design space	*A*	*B*	*AB*	*A* ^2^	*B* ^2^	*F*‐value	Model
Bed bug	< 0.001^a^	< 0.001^a^	0.01^a^	0.001^a^	0.001^a^	66.43	< 0.001^a^
Bed bug egg	< 0.001^a^	< 0.001^a^	0.04^a^	< 0.001^a^	0.008	91.18	< 0.001^a^

^a^Significant model terms.

### 3.5. Effects of Variables on the Mortality of Bed Bug and Bed Bug Egg

The interaction between lethal temperature and time is illustrated using three‐dimensional and two‐dimensional contour plots (Figures 4 and 5). Both plots demonstrate that increasing temperature and exposure time significantly increase bed bug mortality. Superimposing the contour plots in Figure 4 revealed that the optimum mortality rate occurred at 45°C with an exposure time of 60 min. Similarly, superimposing the contours in Figure 5 indicated that the highest mortality rate was achieved at 48.5°C with a 60‐min exposure. Time and temperature are critical factors in determining bed bug egg mortality, with higher values of both leading to greater mortality rates.

FIGURE 4(a) Contour plot illustrating the interactive effect of time and temperature on the percentage mortality of bed bugs at different lethal temperatures and times. (b) 3D plot showing the percentage mortality versus temperature and time.

**Figure:**
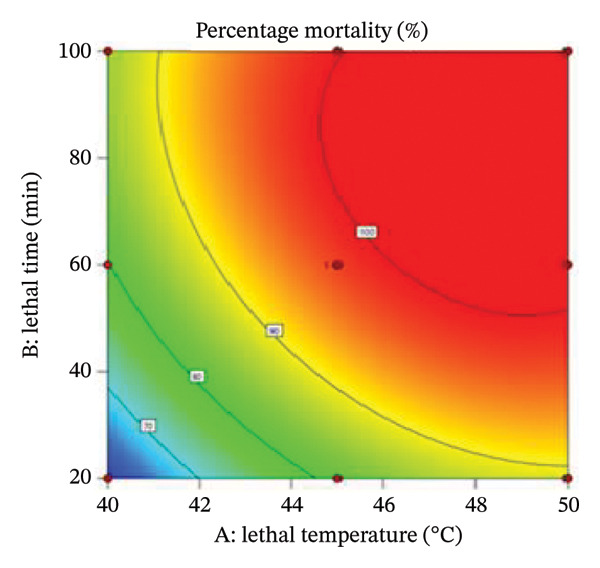
(a)

**Figure:**
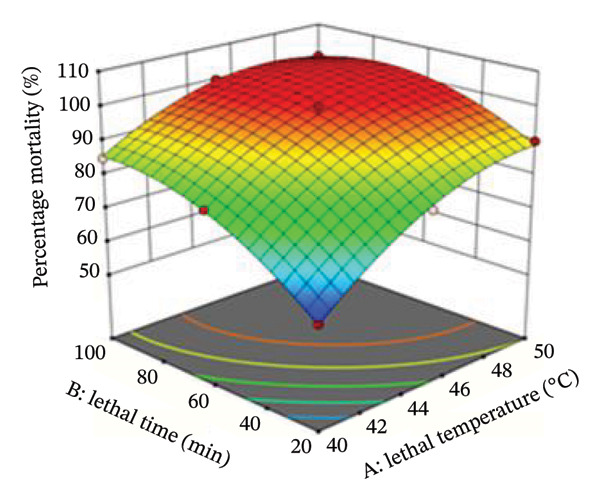
(b)

FIGURE 5Contour plot and 3D plot showing the interactive effect of lethal temperature and lethal time on the mortality of bed bug eggs.

**Figure:**
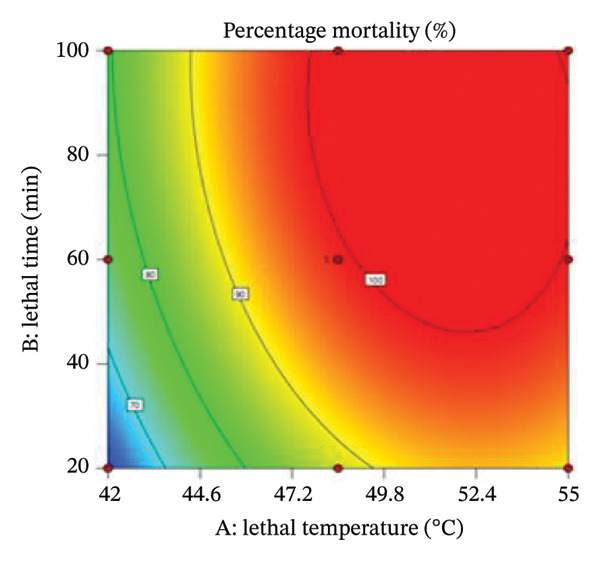
(a)

**Figure:**
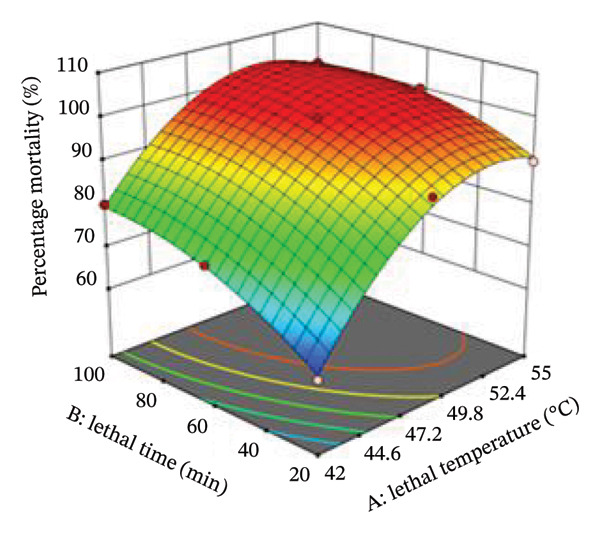
(b)

Figure 6(b) compares the experimental results with the predicted responses, showing a close agreement between the two, indicating that the quadratic model developed in this study adequately represents the experimental data for percentage mortality. The natural logarithm (ln) of the residual sum of squares (SS) plotted against *λ* (Lambda) dips sharply, reaching a minimum near the optimal value of 0.46 (Figure 6(a)). The data do not require transformation because the confidence interval for *λ* includes a value close to this optimal point.

FIGURE 6Relationship between experimental and predicted values calculated using the response surface methodology model based on the Box–Behnken experimental design.

**Figure:**
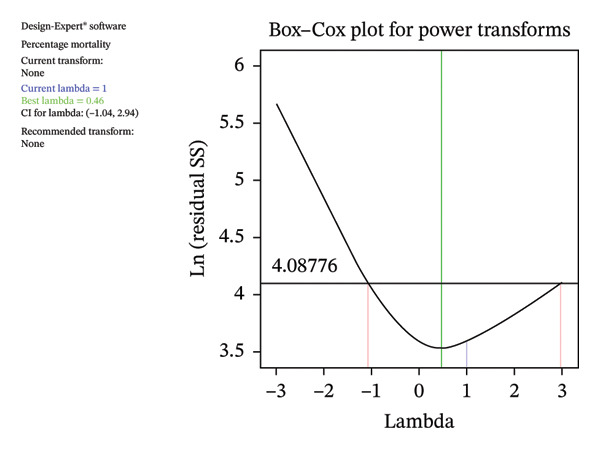
(a)

**Figure:**
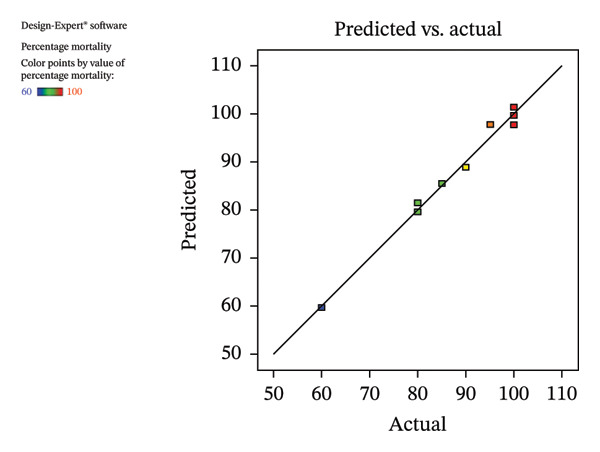
(b)

### 3.6. Percentage Mortality of Bed Bugs and Their Eggs in Cracked Wood

#### 3.6.1. Percentage Mortality of Bed Bug Eggs

According to Figure 7, bed bug egg mortality increased as the exposure time was extended across all heat treatment conditions. The treatments with the lowest percentage mortality were 45°C and 55°C, which resulted in approximately 60% and 70% mortality rates, respectively. In contrast, the treatments at 65°C and 75°C recorded the highest mortality rates, approaching 100%. A clear trend was observed, with higher temperatures leading to greater mortality of bed bug eggs.

FIGURE 7Mortality rate observed on mattress surfaces during localized heat treatment at different exposure times (each experiment used 20 bed bug eggs, *n* = 20).

**Figure:**
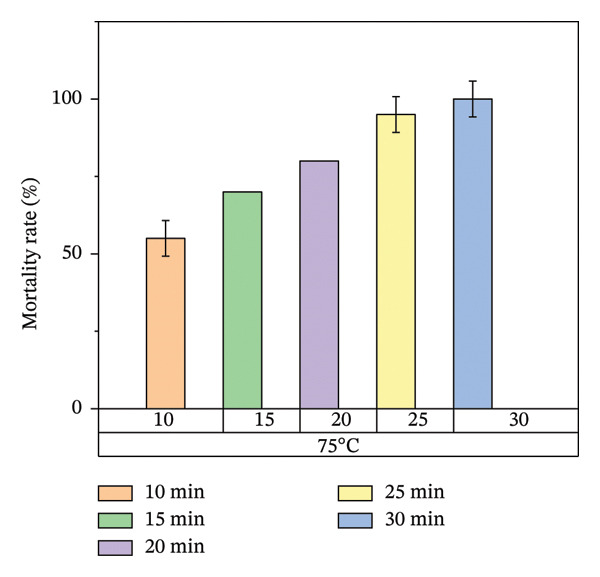
(a)

**Figure:**
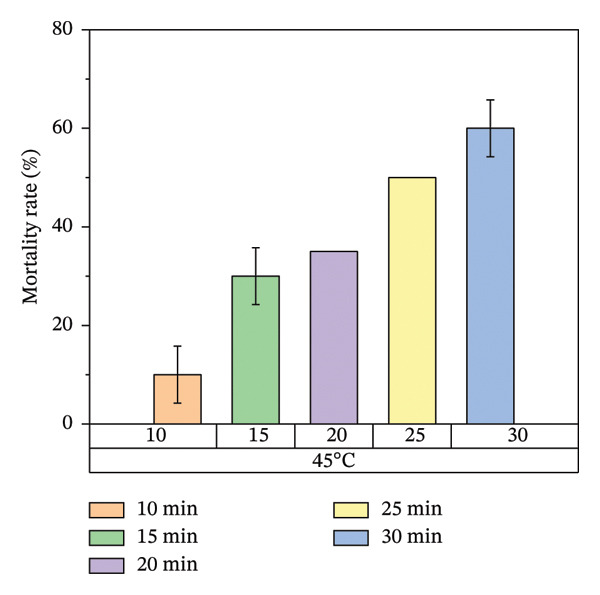
(b)

**Figure:**
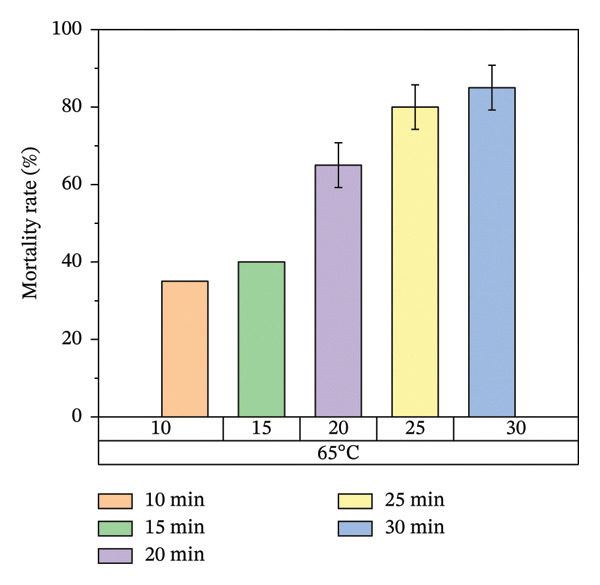
(c)

**Figure:**
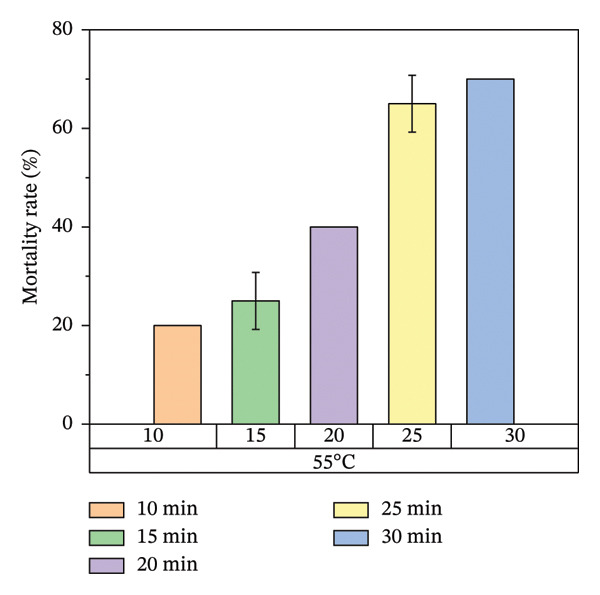
(d)

#### 3.6.2. Percentage Mortality of Bed Bugs

Figure 8 illustrates the results of heat treatment applied to cracked wood surfaces. The percentage mortality of bed bugs increased with temperature, reaching 45% at 45°C, 60% at 55°C, 70% at 65°C, and 100% at 75°C. These findings suggest that the effectiveness of heat treatment is influenced not only by temperature and exposure time but also by the characteristics of the treated surface. For instance, surfaces with cracks or crevices require higher temperatures or longer exposure times to achieve the desired level of bed bug mortality, as shown in Figure 8.

FIGURE 8Mortality rate observed in cracked wood during localized heat treatment at different exposure times (each experiment used 20 bed bugs, *n* = 20).

**Figure:**
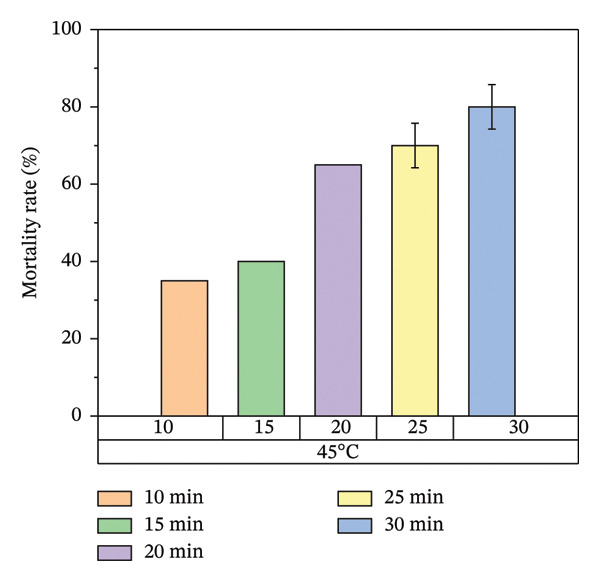
(a)

**Figure:**
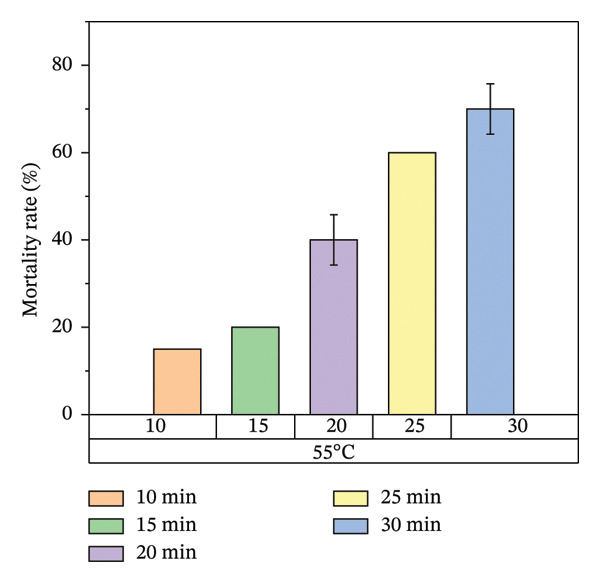
(b)

**Figure:**
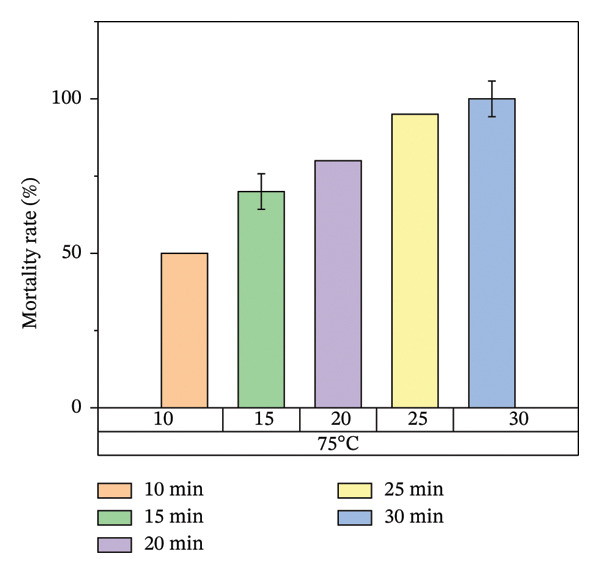
(c)

**Figure:**
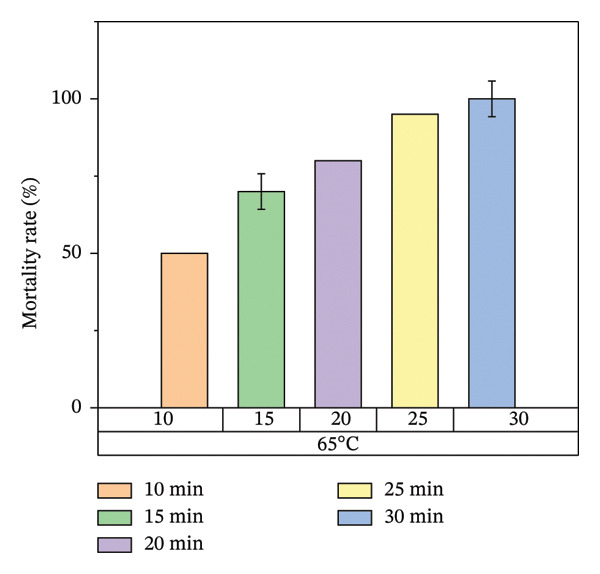
(d)

### 3.7. Percentage Mortality of Bed Bugs and Their Eggs on Mattress Surface

The percentage mortality of bed bugs and bed bug eggs was significantly influenced by the mean mattress temperature. As shown in Table 6, the highest mortality rate (100%) was observed at 75°C. In contrast, 45°C resulted in the lowest percentage mortality among all the heat treatments. Temperatures around 45°C and 55°C showed less pronounced effects compared to 75°C, indicating that higher temperatures are more effective for achieving complete mortality.

**TABLE 6:** Mortality rate of bed bugs and their eggs on mattress surfaces.

Lethal temperature (°C)	Lethal time (min)	Mortality (bed bug) (%)	Bed bug egg mortality (%)
45	10	20 ± 0.00000	20 ± 5.00000
	20	45 ± 11.54701	40 ± 0.00000
	30	60 ± 2.88675	60 ± 2.88675

55	10	40 ± 0.00000	35 ± 5.00000
	20	55 ± 2.88675	45 ± 5.00000
	30	70 ± 0.00000	50 ± 2.88675

65	10	60 ± 2.88675	60 ± 2.88675
	20	75 ± 2.88675	60 ± 8.66025
	30	80 ± 0.00000	75 ± 5.00000

75	10	100 ± 0.00000	90 ± 0.00000
	20	100 ± 0.00000	100 ± 0.00000
	30	100 ± 0.00000	100 ± 0.00000

*Note:* Each experiment used 20 bed bugs, *n* = 20, percentage mortality expressed in mean ± SD.

## 4. Discussion

Developing cost‐effective and practical heat therapies as alternatives to insecticidal treatments for bed bug control requires a comprehensive understanding of the effects of elevated temperatures on these pests. This study aimed to determine the minimal lethal temperature (*L*_Temp_) and the time (*L*_Time_) required to eliminate bed bugs and their eggs effectively.

Our findings revealed that temperatures resulting in 40%–100% knockdown and mortality rates were effective against the selected bed bug species. The temperatures that successfully killed all life stages of the species, in descending order, were 49°C, 47°C, 45°C, 43°C, and 41°C. The optimal temperature for achieving 100% mortality of bed bugs and their eggs was 45°C, with a lethal time of 60 min. Previous research has similarly shown that adult bed bugs can be killed at 45°C, although longer exposure times exceeding 10 min were required.

In cracked wood surfaces, the mortality rate of bed bugs and their eggs was highest at 75°C, with a lethal time of 30 min. Wang and colleagues [8] conducted similar research demonstrating that steam heat can eliminate bed bug infestations in cracked wood surfaces at 75°C with a lethal time of just 8 s. This reduced time was attributed to the direct heating method used in their study [14, 28]. However, in contrast to our findings, a study by Kells and Goblirsch [15] at the University of Minnesota reported that the lethal temperature (*L*_Temp_99) for adults was 48.3°C, while the *L*_Temp_99 for eggs was 54.8°C. Recent reports on heat treatment therapy by various scholars have been presented in Table 7.

**TABLE 7:** Recent reports on heat treatment therapy by various scholars.

Bed bug life span	*L*_Temperature_ (°C)	*L*_Time_	Heat source	Reference
Adult and egg	36–38	1–3 weeks	Climate chambers	[29]
	34–40	3–9 days	Climate chambers	[30]
	27–36	0–14 weeks	Not stated	[31]
	45	60	Oven	Present study

The variations in lethal temperature and lethal time observed in this study may be attributed to altitude differences and local temperature conditions in the study area. Additionally, factors such as exposure duration and the specific temperature levels used to achieve mortality could also influence the outcomes. The underlying mechanism of bed bug mortality at elevated temperatures likely involves a shift in the Michaelis constant, leading to enzyme–substrate dissociation and triggering metabolic failure. Temperature increases also enhance the proportion of molecules with sufficient energy for enzymatically catalyzed reactions [19, 20].

The RSM was utilized to optimize heat treatment parameters. In this study, the response variable was percentage mortality, with lethal time and temperature identified as critical factors [32]. Our findings indicate that a temperature of 45°C and a lethal time of 60 min are optimal for 100% mortality. The RSM model demonstrated a significant influence of both factors, with temperature exerting the greatest impact. This was reflected in the positive coefficients for both variables in the model equation.

The *F*‐values of 66.43 for bed bug mortality and 91.18 for bed bug egg mortality indicate that the model is highly significant, with less than a 0.01% probability of these results being attributed to random noise. Statistically significant *p* values for lethal temperature (*A*), lethal time (*B*), their interaction (*AB*), and quadratic terms (*A* ^2^ and *B* ^2^) further validate the model’s predictive accuracy. The nonsignificant lack of fit suggests that the model adequately represents the experimental data, with a high probability (75.41%) that any deviation is due to noise [33, 34].

Four different lethal temperatures were tested on bed bugs placed on a mattress surface, along with three different lethal times. Bed bugs were placed 2 cm from the mattress edge to address challenges observed during insecticidal treatments. Heat treatments at 45°C and 55°C had a limited impact, resulting in low mortality rates. However, a temperature of 65°C achieved a 90% mortality rate, and full extermination was observed at 75°C maintained for 30 min. This finding highlights the importance of temperature retention on mattress surfaces, as heat tends to remain trapped, facilitating effective extermination.

Figure 7 illustrates the increase in bed bug and egg mortality rates as the temperature spreads across the treated mattress surface. The results align with prior research by Wang et al., which investigated the use of steam heat for bed bug control [13]. Wang’s study found that treating cracked wood surfaces with a temperature of 75°C for 8 s eradicated all bed bugs, including their eggs, due to the direct heating technique employed. Similarly, Kells and Goblirsch [15] reported that lethal temperatures (*L*_Temp_99) for bed bug adults and eggs were 48.3°C and 54.8°C, respectively. The discrepancy with our findings may be attributed to differences in heating methods, exposure times, and environmental conditions.

The challenges associated with heating large items, such as mattresses, include uneven heat distribution and bed bug movement to cooler areas, which can compromise treatment effectiveness. Understanding these dynamics and optimizing treatment protocols are critical for enhancing bed bug control strategies. Additionally, surfaces with cracks and crevices require higher temperatures or longer exposure times to achieve full extermination, as shown by the comparison between the mattress and cracked wood surfaces.

Our findings provide valuable insights into the effectiveness of heat treatment for bed bug control and highlight the importance of substrate type and temperature distribution. However, further validation and optimization of the RSM model are recommended. Future studies could employ advanced statistical techniques, such as fuzzy neural networks or regression analysis models [35–37], to refine predictions and explore interactions between variables (e.g., AB, AC). Additionally, experiments investigating the effects of varying substrate types and environmental conditions will enhance the generalizability of these results.

## 5. Conclusion

In summary, this research demonstrates that using lethal temperature and lethal time is an effective method for pest control. This mechanical approach eradicates pests, such as bed bugs and other harmful insects, without causing damage to materials. Our findings indicate that a temperature of approximately 45°C is ideal for eliminating bed bugs and their eggs, while a higher temperature of 75°C is recommended for treating mattresses and cracked wood surfaces. Currently, many individuals rely on chemical pest control methods, such as insecticides. However, these methods often pose significant drawbacks, including the development of insect resistance and potential health risks to humans. In contrast, our results highlight that using low‐cost equipment to apply lethal temperature and time offers an effective, affordable, and nonchemical alternative to insecticides. This method is particularly advantageous because it can be integrated with other bed bug control strategies without causing significant disruption to treated environments. Additionally, it provides a safer and more sustainable solution compared to traditional chemical treatments, eliminating the health hazards associated with insecticide exposure. To further validate its effectiveness across different habitats and environmental conditions, future studies should explore the application of this technique under various field settings. Ultimately, by uniquely employing response surface optimization to establish tailored heat treatment parameters for challenging substrates like mattresses and cracked wood, this study bridges a critical national knowledge gap and provides a novel, comprehensive framework for environmentally friendly bed bug management.

## Author Contributions

Fitsum Dejene: investigation, methodology, writing–original draft, conceptualization, and formal analysis. Solomon Abera: investigation, methodology, writing–original draft, conceptualization, and formal analysis. P. R. Jeyaramraja: investigation, methodology, writing–original draft, conceptualization, and formal analysis. Yonas Syraji: investigation, writing–original draft, methodology, writing–review and editing, and formal analysis.

## Funding

No funding was received for this study.

## Ethics Statement

The authors have nothing to report.

## Consent

The authors have nothing to report.

## Conflicts of Interest

The authors declare no conflicts of interest.

## Data Availability

All sources of the data are available in the manuscript.
